# A Blue Light-Inducible CRISPR-Cas9 System for Inhibiting Progression of Melanoma Cells

**DOI:** 10.3389/fmolb.2020.606593

**Published:** 2020-11-19

**Authors:** Xia Wu, Haiyan Huang, Bo Yu, Jianzhong Zhang

**Affiliations:** ^1^Department of Dermatology, Peking University People’s Hospital, Beijing, China; ^2^Department of Dermatology, Skin Research Institute of Peking University Shenzhen Hospital, Peking University Shenzhen Hospital, Shenzhen, China

**Keywords:** melanoma, light-inducible system, CRISPR/Cas9, gene switch, BRAF V600E

## Abstract

Melanoma is an aggressive skin tumor that shows a high mortality rate and level of metastasis. BRAF gene mutation (BRAF V600E) is directly related to the occurrence of melanoma. In this study, a light-inducible gene expression system was designed to control the Cas9 transcription, which could then cleave the BRAF V600E. To prove the potential utility of this system in melanoma, the physiological function of melanoma cells was tested. It illustrated that the light-induced CRISPR-Cas9 system could inhibit the progression of G361 and A375 cells. Thus, this system may provide a novel therapeutic strategy of melanoma intervention.

## Introduction

Melanoma is one of the most malignant skin tumors. Despite its low prevalence rate, its incidence increases every year (Hernandez-Davies et al., 2015; Chang et al., 2020; Strub et al., 2020). For more than 40 years, there have been few available treatments, and none of the clinical trials conducted during this period have been successful (Schadendorf et al., 2018). *BRAF V600E* was found as the most common mutant position and had a strong connection with the morbidity and prognosis of melanoma (Young et al., 2012; Greaves et al., 2013). Over the past years, molecule-targeted drugs – such as BRAF and MEK inhibitors have been used in clinical practice (Luke et al., 2017; Dummer et al., 2018a,b; Chavda and Bhatt, 2020). Though effective initially, drug resistance often occurs after treatment for 2–18 months (Chang et al., 2020). As the resistance developed, the tumor cells almost always become more invasive to further dissemination and metastasis (Cohen-Solal et al., 2018). To understand the underlying mechanism of this resistance, previous researchers found the reactivation of the BRAF/MEK/ERKs pathway accounted for 80% of the resistant tumors (Rizos et al., 2014). In addition, the PI3-K/AKT pathway that is closely interacted with the BRAF/MEK/ERKs could be another cause of drug resistance when reactivated (Davies, 2012; Rizos et al., 2014).

CRISPR-Cas9 was used to edit the genome in various fields (Carey and Gagnon, 2020; Crunkhorn, 2020; Moghadam et al., 2020). Emerging evidence suggests that genome editing of BRAF V600E is possible and effectively induces the apoptosis of melanoma cells (Yang et al., 2017). Our group has tried to design sgRNAs targeting BRAF V600E and edited it with the combined effect of Cas9. According to the results of Sanger sequencing and cell function tests, the edited BRAF V600E caused the apoptosis of melanoma cells. Although CRISPR-Cas9 is efficient, how to precisely control its function requires further investigation (Akcakaya et al., 2018; Lino et al., 2018; Huang et al., 2020). Thus, developing a more accurate regulatory system is in urgent need. As an epidermal tumor, melanoma is easily accessible to light, which is widely used in various disciplines as a gene regulatory tool, especially in synthetic biology (Yazawa et al., 2009; Kennedy et al., 2010; Rana and Dolmetsch, 2010; Lin et al., 2016). According to a previous study, a blue light-switchable transgene system (GAVPO) was presented and tested in mammalian cells and in mice (He et al., 2020). We considered applying this system to the treatment of melanoma.

Here, we described a light-inducible gene expression system based on CRISPR-Cas9 and the optimized light-switchable device (GAVPO) interaction. The GAVPO system consists of three parts: GAL4 (65), Vivid protein and p65 (Wang et al., 2012). The GAL4(65), which contains GAL4 residues 1–65, virtually eliminates binding to its consensus cognate DNA sequence, the upstream activating sequence of Gal (UASG). Vivid (VVD) protein, the smallest light-oxygen-voltage (LOV) domain–containing protein, forms a rapidly exchanging dimer upon blue-light activation. The transcriptional activator p65 was ligated to the C-terminal of GAL4 (65)-VVD fusion protein to produce the GAVP system. Then, the double mutations of C71V and N56K in the VVD domain decreased the background gene expression to a minimal level [optimized GAVP (GAVPO)], which was named the light-on system (Light On). When exposed to blue light, GAVPO binds to the UASG and activates downstream gene expression. In this study, we made a codon optimization of the original GAVPO sequence and combined the light-on system with CRISPR and applied it to melanoma, which was a controllable and efficient anti-tumor method. When the GAVPO-CRISPR system was transferred into melanoma cells, under the effect of blue light, the proliferation, invasion and migration of melanoma cells were inhibited, and the apoptosis rate was selectively increased. As a simple method, the light-induced tumor-killing module has worked effectively, and it was expected to become a novel anti-cancer method.

## Results

### CRISPR-Cas9 System Cleaved the BRAF V600E

The function and specificity of our sgRNA were tested by gRNA Activity Assay Test Kit (Beijing Syngentech Co., Ltd.). The mKate gene in pHS-ACR-ZQ191 and pHS-ACR-ZQ190 was terminated prematurely by a terminator. In order to test the activity of our gRNA, the target mutant BRAF was inserted after the terminator. After the action of Cas9 and sgRNA, the double-strand DNA at the target site was cleaved to form a double-strand break (DSB), and the mKate was activated through cell homologous recombination. HEK293 cells were transfected with pHS-ACR-ZQ170 and pHS-AVC-ZQ190 as a negative control group (NC group) and transfected with pHS-ACR-ZQ170 and pHS-AVC-ZQ191 as the treatment group. The gRNA activity assay demonstrated that the CRISPR-Cas9 system specifically cleaved the mutant BRAF V600E (Supplementary Figure S1), but had no effect on the wild type.

**FIGURE 1:**
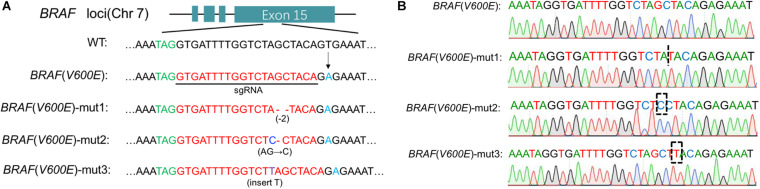
Cas9 edited BRAF V600E. **(A)** Specific sgRNA was designed for BRAF locus. Under the action of Cas9/sgRNA, the BRAF V600E sequence was cut in melanoma cells. Here three examples are cited. **(B)** Detailed sequencing maps.

The Cas9-sgRNA (BRAF V600E) plasmid was transferred to A375 and G361 cells, and the cell genome was extracted and amplified by Polymerase Chain Reaction (PCR). The amplified DNA fragments were cloned by TA cloning technology. Twenty-one single positive clones were picked randomly and the plasmids were extracted for sequencing. According to the results, we found that BRAF V600E was successfully cut out and three examples were shown (Figure 1).

### The Blue Light-Inducible BRAF V600E Gene Cleavage System Was Designed and Constructed

**FIGURE 2:**
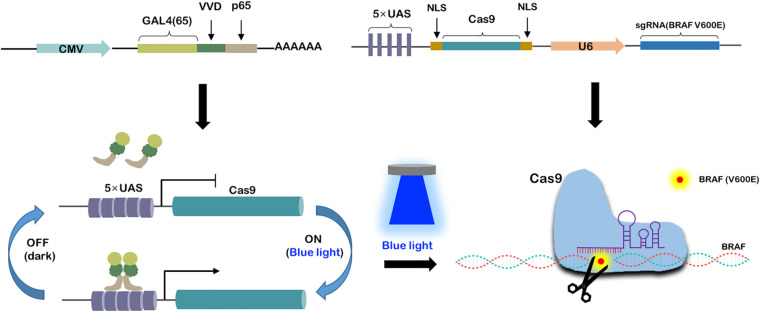
Construction of blue light-induced BRAF V600E cleavage system. Synthesis of a blue light-induced BRAF V600E cleavage system based on two plasmids. One is CMV promoted GAL4(65), VVD and p65 fusion protein. The other is 5× UAS promoted Cas9 and U6 promoted sgRNA. Under blue light condition, the fusion proteins get dimerized and activated the 5× UAS promoter, leading to gRNA-Cas9 expression and BRAF V600E specific editing.

As shown in Figure 2, we have constructed two vectors to form a blue light-induced BRAF V600E cleavage system. The GAVPO element driven by CMV promoter was a fusion protein composed of GAL4(65), VVD and p65. The Cas9 gene was promoted by 5 × UAS and sgRNA targeting BRAF V600E was promoted by a U6 promoter. The DNA-binding property of Gal4(65)-VVD-p65 fusion protein would be light-switchable. As the light induced dimerization of the fusion protein, it could bind to the UAS and activate Cas9 transcription. Therefore, blue light could flexibly regulate the work of Cas9-sgRNA systems. In this study, we call this system the “GAVPO-CRISPR.”

### Cell Proliferation Was Inhibited by the GAVPO-CRISPR System in Melanoma Cells

**FIGURE 3:**
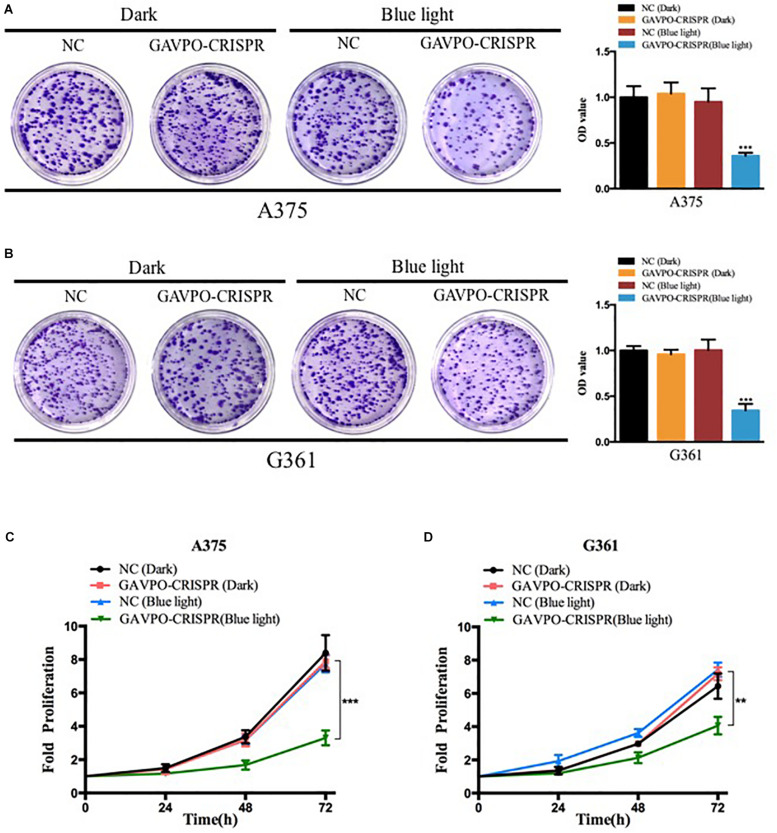
Light-induced GAVPO-CRISPR system inhibited melanoma cell growth. **(A,B)** Colony-formation assay implicating the effect of light-induced GAVPO-CRISPR system under blue-light or dark conditions on cell proliferation in A375 **(A)** and G361 **(B)** cells, compared to the negative control. **(C,D)** CCK-8 assay representing the effect of light-induced GAVPO-CRISPR system under blue-light or dark conditions on cell proliferation in A375 **(C)** and G361 **(D)** cells, compared to the negative control. Data are presented as the means ± SD from at least three biological replicates. (*P < 0.05, **P < 0.01, ***P < 0.001).

To examine the effects of the GAVPO-CRISPR system on melanoma cell growth under the illumination condition (0.84 W/m^2^; 1 s:30 s), the cell counting kit-8 (CCK8) assay was performed. The results showed that the GAVPO-CRISPR system suppressed cell growth in A375 (Figure 3C) and G361 (Figure 3D) significantly (*P* < 0.01) after 72-h exposure to the blue light, compared to the negative controls. The colony-formation assay investigated further into the effect of GAVPO-CRISPR on the proliferation of melanoma cells (Figures 3A,B), and its results supported the CCK-8 experiment. After a 2-week cell culture, the picture of the cells stained with crystal violet solution directly reflects the inhibitory effect of light-induced GAVPO-CRISPR system on the growth of melanoma cells.

### Cell Migration Was Suppressed by the GAVPO-CRISPR System in Melanoma Cells

**FIGURE 4:**
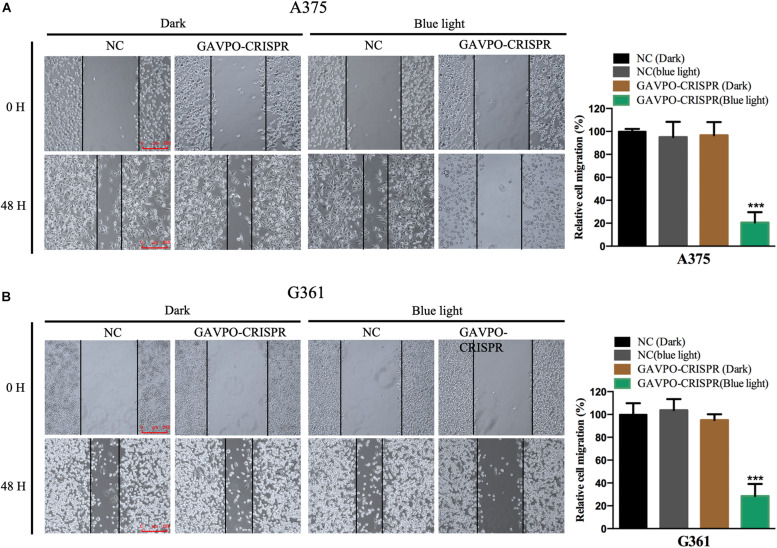
Light-induced GAVPO-CRISPR system inhibited melanoma cell migration. **(A)** Wound healing assay implicating the effect of light-induced GAVPO-CRISPR system under blue-light or dark conditions on cell migration in A375 cells, compared to the negative control. **(B)** Wound healing assay representing the effect of light-induced GAVPO-CRISPR system under blue-light or dark conditions on cell migration in G361 cells, compared to the negative control. Data are presented as the means ± SD from at least three biological replicates. (*P < 0.05, **P < 0.01, ***P < 0.001).

To investigate the effects of the GAVPO-CRISPR system on melanoma cell migration under the blue light (0.84 W/m^2^; 1 s:30 s), the wound healing assay was performed. Blue light and dark conditions were given when scratches were formed, and the healing of scratches was observed after 48 h. The results showed that GAVPO-CRISPR could inhibit the migration of A375 and G361 cells under blue light illumination (Figures 4A,B).

### Cell Invasion Was Restrained by the GAVPO-CRISPR System in Melanoma Cells

**FIGURE 5:**
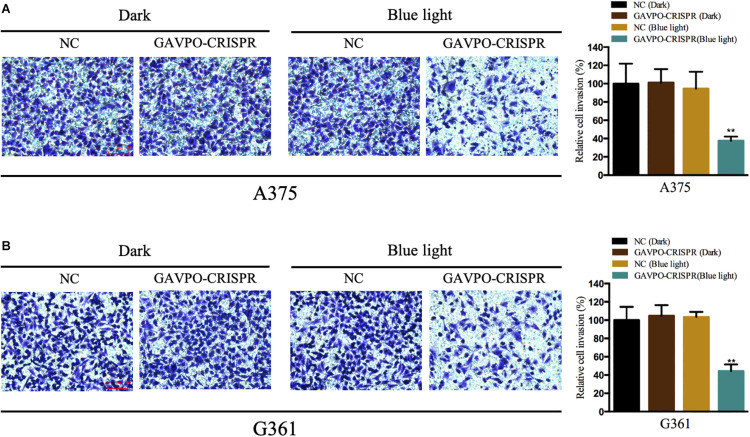
Light-induced GAVPO-CRISPR system restricted melanoma cell invasion. **(A)** Transwell assay implicating the effect of light-induced GAVPO-CRISPR system under blue-light or dark conditions on cell invasion in A375 cells, compared to the negative control. **(B)** Transwell assay representing the effect of light-induced GAVPO-CRISPR system under blue-light or dark conditions on cell invasion in G361 cells, compared to the negative control. Data are presented as the means ± SD from at least three biological replicates. (*P < 0.05, **P < 0.01, ***P < 0.001).

Transwell assay was performed to assess the effect on cell invasion of GAVPO-CRISPR treated melanoma cells. After 24 h of dark and blue light (0.84 W/m^2^; 1 s:30 s) conditions, the cell invasion rate was obviously suppressed in the GAVPO-CRISPR group compared to the groups under dark conditions and the control group under blue light (Figures 5A,B). These results demonstrated that the light-controlled system could restrain cell invasion in melanoma cells.

### Cell Apoptosis Was Promoted by the GAVPO-CRISPR System in Melanoma Cells

**FIGURE 6:**
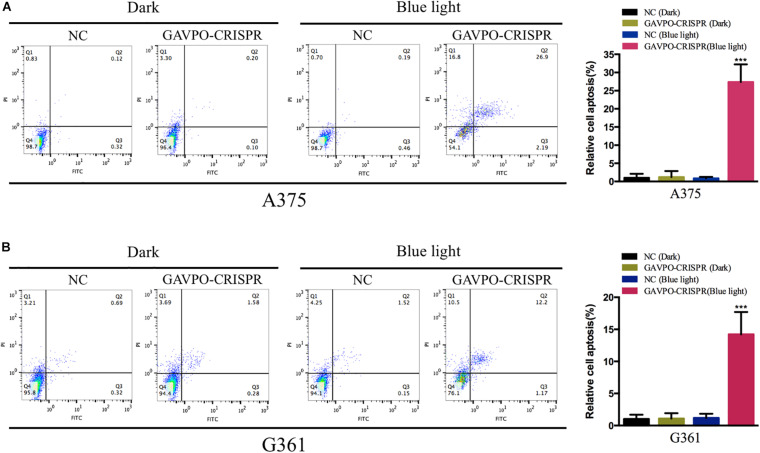
Light-induced GAVPO-CRISPR system promoted melanoma cell apoptosis. **(A)** Flow cytometry results representing the effect of light-induced GAVPO-CRISPR system under blue-light or dark conditions on cell apoptosis in A375 cells, compared to the negative control. **(B)** Flow cytometry results representing the effect of light-induced GAVPO-CRISPR system under blue-light or dark conditions on cell apoptosis in G361 cells, compared to the negative control. Data are presented as the means ± SD from at least three biological replicates. (*P < 0.05, **P < 0.01, ***P < 0.001).

Flow cytometry assay was performed to detect the effects on cell apoptosis of GAVPO-CRISPR treated melanoma cells. Before the flow cytometry, cells were cultured in dark and blue light (0.84 W/m^2^; 1 s:30 s) condition for 48 h. As shown in Figures 6A,B, compared to the negative controls, the apoptosis of A375 and G361 in the GAVPO-CRISPR group was significantly increased. (^∗∗∗^*P* < 0.001). These results demonstrated that the light-controlled system could promote cell apoptosis in melanoma cells.

## Discussion

Melanoma is a highly malignant skin cancer that is commonly treated with surgical resection and chemotherapy. Early stage patients benefit from the surgery, yet the prognosis of the late-stage patients is poor. Tumorigenesis is a result of accumulated multi-gene mutation, in which BRAF gene mutation (mainly BRAF V600E) is significantly higher than other pathogenic genes in melanoma. It is therefore one of the research hotspots in melanoma therapy.

In the field of gene therapy, CRISPR is a convenient and effective technology. Using CRISPR to edit the mutation sites of genes can effectively disrupt the function of pathogenic genes (Yang et al., 2017; György et al., 2019; Wang D. et al., 2019; Zhao H. et al., 2020). In this study, we used CRISPR-Cas9 technology to edit the BRAF V600E gene and found a good inhibitory effect on tumor cells. Since melanoma is often found on the skin surface with a limited range of size. It is meaningful to control CRISPR in time and space dimensions.

As a simple and easy-to-obtain light source, blue light is often used for anti-inflammation and sterilization on the skin, without side effects on the human body (Lotan et al., 2019; Zhao J. et al., 2020). In this study, we used blue light as the controller of the CRISPR-Cas9 system. In the absence of blue light, the CRISPR system stopped working. When irradiated by blue light, CRISPR-Cas9 was activated to edit the gene. By controlling blue light exposure, the CRISPR-Cas9 tool can be flexibly regulated in the time and space dimensions, making it an ideal tool for anti-BRAF V600E.

In this study, we cultured cells under dark and 460 nm blue light conditions, respectively, and verified their functions through a series of cell functional experiments. The results showed that blue light could effectively activate the function of GAVPO-CRISPR and inhibit the proliferation of tumor cells.

In this study, melanoma mutant gene BRAF V600E was edited by the CRISPR gene-editing tool, resulting in an obvious killing effect on tumor cells. Then, combined with the characteristics of melanoma, the blue light on the surface of the skin can be used to control the work of CRISPR manually. This study expands the application scenario of the CRISPR tool and exerts its anti-tumor effect by effectively combining it with a photogenetic tool. In clinical gene therapy, we can further optimize the system, such as using smaller Cas protein (like CasX) to play the role of gene editing, and using vectors such as adeno-associated virus (AAV) to deliver them to organisms for cancer biotherapy. In fact, researchers have made many successful attempts in the treatment of diseases with light control devices. The research of AAV has entered the clinical treatment stage, the combination of AAV and light control devices is feasible (Wang E. et al., 2019; Yang et al., 2020; Yu et al., 2020).

In summary, we have synthesized a gene circuit that combined photogenetic tools and CRISPR gene editing techniques to specifically suppress the melanoma cells. Our study explores a new approach for the potential treatment of melanoma, and the synthetic gene circuit has the potential for clinical application.

## Materials and Methods

### Plasmids Construction

To construct the vector expressing the Cas9 and sgRNA targeting BRAF V600E, the three sgRNAs were designed and synthesized by Syngentech Co., Ltd. (Beijing, China). To generate a plasmid that expresses sgRNA targeting mutant BRAF in melanoma cells, a plasmid (pHS-ACR-ZQ170) containing a U6 promoter driven sgRNA that targeted the mutant BRAF gene and a Cas9 protein driven by phEF1a promoter was constructed. In order to test the function and specificity of the sgRNA, two plasmids containing a wild-type BRAF sequence (pHS-ACR-ZQ190) and BRAF V600E (pHS-ACR-ZQ191) were constructed. Both plasmids contained a prematurely terminated mKate gene (a new type of dark red fluorescent protein originated from TagRFP) driven by phEF1a promoter. After the single-strand annealing test, an effective sgRNA was picked out and used.

To construction the vector that expresses the GAL4(65)-VVD-p65, the sequence of GAL4(65)-VVD-p65 was inserted into pHS-AVC-LW477 between the restriction sites *Eco*RI and *Bam*HI. To create vectors expressing 5× UAS-Cas9-U6-sgRNA (BRAF V600E), we used the UAS sequence to replace CMV promoter, respectively and place the sequence of Cas9 in the middle of sgRNA and UAS in the pHS-ACR-LW352 vector. Finally, the ultimate two vectors (GAL4(65)-VVD-p65 and UAS-Cas9-sgRNA) were packaged by lentivirus. Relative sequences were presented in Supplementary Table S1 and Supplementary Figure S1.

### Cell Lines and Cell Culture

The melanoma cell lines (A375 and G361) were purchased from the Institute of Cell Research, Chinese Academy of Sciences, Shanghai, China. The A375 and G361 cells were maintained in 1640 medium (Invitrogen, Carlsbad, CA, United States) with 10% fetal bovine serum (FBS) and 1% antibiotics (100 ug/ml streptomycin and 100 U/ml penicillin) at 37°C in the atmosphere of 5% CO_2_.

### Cell Transfection and Illumination

The constructed vectors from *Escherichia coli* bacteria were extracted by E.Z.N.A Fastfiler Endo-free Plasmid Maxiprep kits (Omega, Norcross, GA, United States). The cell was transfected with vectors using Lipofectamine 3000 Transfection Reagent (Invitrogen, Carlsbad, CA, United States) according to the manufacturer’s protocol. After transfection in dark about 12 h, cells were illuminated by a blue light LED lamp (460 nm, 0.84 W m^–2^). The lentivirus vectors were packaged and concentrated by Syngentech Co., Ltd. (Beijing, China). After puromycin screening, cells were illuminated by blue light. The blue light can be controlled by a timer adjusting the illuminate dose.

### DNA Extraction, Original TA Cloning and Gene Sequencing

The DNA Maxi Kit (Omega, Norcross, GA, United States) was used to extract cell DNA after transfection according to the manufacturer’s protocol. The primers were used to amplify the sequence containing BRAFV600E by PCR, then the PCR fragments were sited into the pMDTM19-T vector using pMDTM19-T Vector Cloning Kit (Takara, Dalian, China). The primers used were:

BRAFV600E-forward: GCTGTGGATCACACCTGCCTT AAA
BRAFV600E-reverse: TCGCCCAGGAGTGCCAAGAGA

The cloning vectors were sequenced by Syngentech Co., Ltd. (Beijing, China).

### Wound Healing Assay

The wound healing assay was used to assess the migration ability of G361 and A375 *in vitro*. Before a blue light illumination, lentivirus infected cells were seeded in the 6-well plate to get more than 90% confluence and serum-starved for 24 h. Then illumination started, wounds were scratched by passing the sterile 200 μl pipette tip across the monolayer cells at the same time. We considered the time of wound infliction as 0 h, and wound closure was determined at 24 h. The width of the wound was monitored with the help of a digital camera system and the areas covered by migrated cells (%) were quantified.

### Cell Proliferation Assay

The effects of dark and blue light illumination to melanoma cells were measured by CCK-8 assay. After dark and illumination treating, about 3 × 10^3^ cells per well were seeded in a 96-well plate and pre-incubated for 12 h. At the time of 0, 24, 48, and 72 h, 10 μl of Cell Counting Kit solution (TransGen, Beijing, China) was added to each well. The absorbance at 450 nm was calculated by the CCK-8 reader machine (Bio-Rad, Hercules, CA, United States) after an hour’s incubation. The experiments were performed at least three times. For the colony-formation assay, the transfected G361 and A375 cells were cultured in 6 cm culture dishes at a density of 2000 cells per well and incubated for 15 days and exposed to blue light. Finally, the cells were stained with 0.1% crystal violet and imaged. The stained cells were washed with 33% glacial acetic acid. The absorbance in each well was measured at 550 nm using a microplate reader.

### Cell Invasion Assay

The effects of dark and blue light illumination on cell invasion were measured by Transwell assay. G361 and A375 cells were seeded in 24-well plates. About 5 × 10^5^ cells were seeded in triplicate onto a 24-well plate with chambers (Corning, NY, United States) coated with Matrigel. The lower chamber contained 10% FBS medium and the upper one contained a serum-free medium. With the incubation of 24 h, we counted the percentage of cells passing the membranes under a microscope.

### Flow Cytometry Assay

G361 and A375 cells were seeded in six-well plates. Then transfected cells were collected after incubation under light/dark for 24 h and harvested using trypsin without EDTA. According to the manufacturer’s protocols, the FITC Annexin V Apoptosis Detection Kit (TransGen, Beijing, China) could double stain cells with FITC Annexin and PI. The ratio of early apoptotic cells in melanoma cells was determined by the flow cytometry (EPICS, XL-4, Beckman, CA, United States). The experiments were done at least three times.

### Statistical Analyses

All data from at least three independent experiments were presented as mean ± standard deviation (SD). Statistical data was analyzed by SPSS 20.0 software (SPSS Inc., Chicago, IL, United States). *P* < 0.05 was considered statistically significant.

## Data Availability Statement

All datasets presented in this study are included in the article/Supplementary Material.

## Author Contributions

XW and HH performed the experiments and data analysis. XW prepared all the figures and wrote the manuscript. JZ and BY designed and supervised the project. All authors read and approved the final manuscript.

## Conflict of Interest

The authors declare that the research was conducted in the absence of any commercial or financial relationships that could be construed as a potential conflict of interest.
