# Supplementary material for: A Blue Light-Inducible CRISPR-Cas9 System for Inhibiting Progression of Melanoma Cells
Source: Front Mol Biosci. 2020 Nov 19;7:606593. doi: 10.3389/fmolb.2020.606593 (PMC7710612; doi:10.3389/fmolb.2020.606593)

**A blue light-inducible CRISPR-Cas9 system for inhibiting progression of melanoma cells**

Xia Wu et al.

**Supplementary Information**

Supplementary Table 1

| Name | cDNA sequence |
| --- | --- |
| sgRAN(V600E) | GTGATTTTGGTCTAGCTACA |
| pHS-AVC-ZQ190 sequence（BRAF） | GTGATTTTGGTCTAGCTACAGTG |
| pHS-AVC-ZQ191 sequence（BRAF V600E） | GTGATTTTGGTCTAGCTACAGAG |
| 5 ×UAS | CGGAGTACTGTCCTCCGAGCGGAGTACTGTCCTCCGAGCGGAGTACTGTCCTCCGAGCGGAGTACTGTCCTCCGAGCGGAGTTCTGTCCTCCG |
| Optimized GAVPO Sequence | ATGAAGCTACTGTCTTCTATCGAACAAGCATGCGATATTTGCCGACTTAAAAAGCTCAAGTGCTCCAAAGAAAAACCGAAGTGCGCCAAGTGTCTGAAGAACAACTGGGAGTGTCGCTACTCTCCCAAAACCAAAAGGTCTCCGCTGACTAGGGCACATCTGACAGAAGTGGAATCAAGGCTAGAAAGACTGGAAAGAAGCATCGCCACCAGGAGCCACACACTGTACGCCCCCGGCGGCTACGATATCATGGGCTACCTGATCCAGATCATGAAGAGGCCTAACCCCCAGGTGGAGCTGGGCCCCGTGGACACAAGCGTGGCCCTGATCCTGTGCGACCTGAAGCAGAAGGATACCCCCATCGTGTACGCCAGCGAGGCCTTTCTGTACATGACCGGCTACTCCAATGCCGAGGTGCTGGGCAGAAATTGCAGATTTCTGCAATCCCCCGATGGCATGGTGAAGCCCAAGTCCACAAGGAAGTACGTGGACAGCAACACCATCAACACCATGAGGAAGGCCATCGACAGGAATGCCGAGGTCCAGGTGGAGGTGGTGAACTTTAAGAAGAACGGCCAGAGGTTTGTGAACTTCCTGACCATGATCCCTGTGAGGGATGAGACCGGCGAGTACAGATACAGCATGGGCTTTCAGTGTGAGACAGAGCTGCAATACCCCTACGACGTGCCTGATTACGCCGAGTTTCAGTACCTGCCTGACACCGACGACAGGCACAGGATCGAGGAGAAGAGAAAGAGGACCTACGAGACATTCAAGAGCATCATGAAGAAGAGCCCCTTCAGCGGCCCTACAGATCCTAGACCCCCCCCTAGAAGGATCGCCGTGCCCAGCAGATCCAGCGCCAGCGTGCCCAAGCCCGCCCCTCAACCATACCCTTTCACCTCCAGCCTGTCCACAATCAACTACGATGAGTTCCCTACCATGGTGTTCCCTAGCGGCCAGATCTCCCAGGCCAGCGCCCTGGCTCCCGCTCCTCCTCAAGTGCTGCCTCAGGCCCCCGCCCCTGCTCCTGCTCCTGCCATGGTGAGCGCCCTGGCCCAGGCTCCAGCCCCTGTTCCTGTGCTGGCCCCCGGACCACCTCAGGCTGTGGCTCCTCCTGCCCCCAAGCCTACACAGGCCGGCGAGGGAACCCTGAGCGAGGCTCTGCTGCAACTGCAATTTGATGACGAGGACCTGGGCGCCCTGCTGGGCAACAGCACCGATCCTGCCGTGTTTACAGACCTGGCCTCCGTGGATAACTCCGAGTTCCAGCAGCTGCTGAACCAGGGCATCCCTGTGGCCCCTCACACAACAGAGCCTATGCTGATGGAGTACCCTGAGGCCATCACAAGACTGGTGACAGGCGCCCAGAGACCTCCCGATCCCGCCCCTGCACCCCTGGGAGCTCCAGGACTGCCCAATGGCCTGCTGTCCGGCGATGAGGACTTTTCCAGCATCGCCGACATGGACTTTTCCGCCCTGCTGTCCCAGATCAGCAGCGATTACAAGGATGACGACGATAAG |
| Original GAVPO Protein | RSIATRSHTLYAPGGYDIMGYLIQIMKRPNPQVELGPVDTSVALILCDLKQKDTPIVYASEAFLYMTGYSNAEVLGRNCRFLQSPDGMVKPKSTRKYVDSNTINTMRKAIDRNAEVQVEVVNFKKNGQRFVNFLTMIPVRDETGEYRYSMGFQCETELQYPYDVPDYAEFQYLPDTDDRHRIEEKRKRTYETFKSIMKKSPFSGPTDPRPPPRRIAVPSRSSASVPKPAPQPYPFTSSLSTINYDEFPTMVFPSGQISQASALAPAPPQVLPQAPAPAPAPAMVSALAQAPAPVPVLAPGPPQAVAPPAPKPTQAGEGTLSEALLQLQFDDEDLGALLGNSTDPAVFTDLASVDNSEFQQLLNQGIPVAPHTTEPMLMEYPEAITRLVTGAQRPPDPAPAPLGAPGLPNGLLSGDEDFSSIADMDFSALLSQISSDYKDDDDK |
| Optimized GAVPO Protein | RSIATRSHTLYAPGGYDIMGYLIQIMKRPNPQVELGPVDTSVALILCDLKQKDTPIVYASEAFLYMTGYSNAEVLGRNCRFLQSPDGMVKPKSTRKYVDSNTINTMRKAIDRNAEVQVEVVNFKKNGQRFVNFLTMIPVRDETGEYRYSMGFQCETELQYPYDVPDYAEFQYLPDTDDRHRIEEKRKRTYETFKSIMKKSPFSGPTDPRPPPRRIAVPSRSSASVPKPAPQPYPFTSSLSTINYDEFPTMVFPSGQISQASALAPAPPQVLPQAPAPAPAPAMVSALAQAPAPVPVLAPGPPQAVAPPAPKPTQAGEGTLSEALLQLQFDDEDLGALLGNSTDPAVFTDLASVDNSEFQQLLNQGIPVAPHTTEPMLMEYPEAITRLVTGAQRPPDPAPAPLGAPGLPNGLLSGDEDFSSIADMDFSALLSQISSDYKDDDD |

**
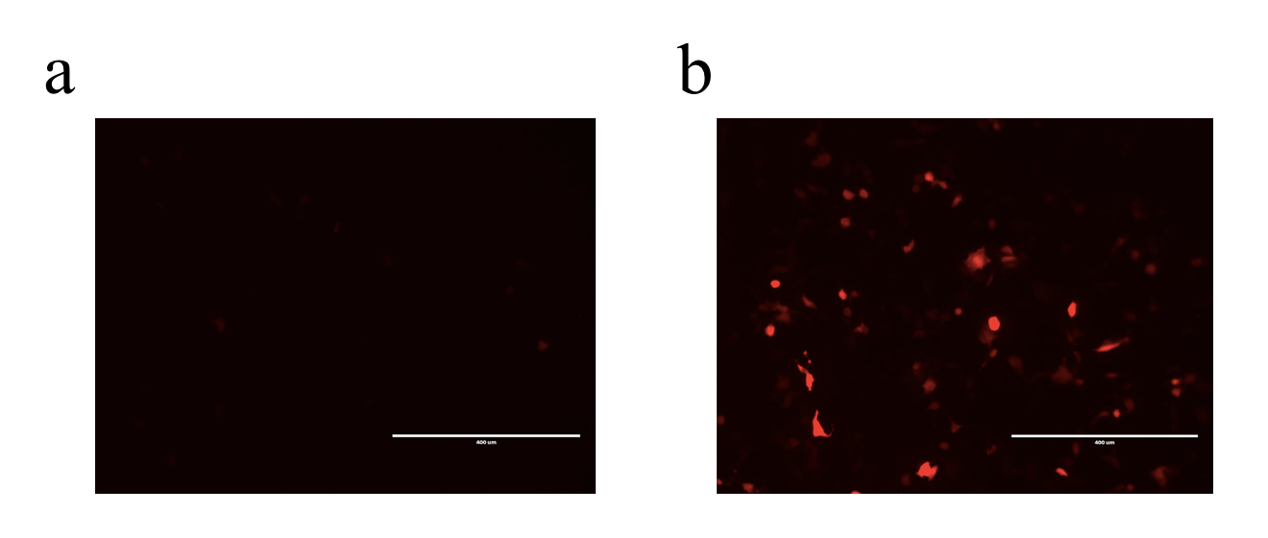
**

**Supplementary Figure.1 Functional test of sgRNAs in HEK293.** The activity of the fluorescent protein was determined by fluorescence microscopy, which indirectly determine the activity and knockout efficiency of the gRNA. Results showed that red fluorescence can only be found in (b) through fluorescence microscopy which meant pHS-ACR-ZQ170 plasmid could cleave mutant target plasmid pHS-AVC-ZQ191 while it could not cleave wild-type target plasmid pHS-AVC-ZQ190.

**Supplementary Figure.2 Maps of plasmids used in this study.**

| pHS-ACR-ZQ170 | pZDonor_Seq1-U6-BRAF(V600E)sgRNA-hEF1a-hCas9-2A-EGFP-2A-Puro-Seq2 |
| --- | --- |
| pHS-AVC-ZQ190 | pZD_Seq1-hEF1a-tmKate-BRAF(V600E)_sgRNA_target1 sequence-tmKate-Seq2 |
| pHS-AVC-ZQ191 | pZD_Seq1-hEF1a-tmKate-BRAF(V600E)_sgRNA_target2 sequence-tmKate-Seq2 |
| pHS-AVC-LW677 | pLV-CMV-GAL4(65)-VVD-p65-IRES-Puro |
| HS-LV005 | pLV-hef1a-MCS-3Xflag-IRES-Puro |
| pHS-ACR-LW352 | pLV-U6-BRAF(V600E) sgRNA-sgRNA_backbone-5XUAS-NLS-cas9-NLS-T2A-bla |
| HS-LV018 | pLV-U6-Bsa I-LacZ-Bsa I-gRNA backbone-CMV-hCas9-2A-Bla |


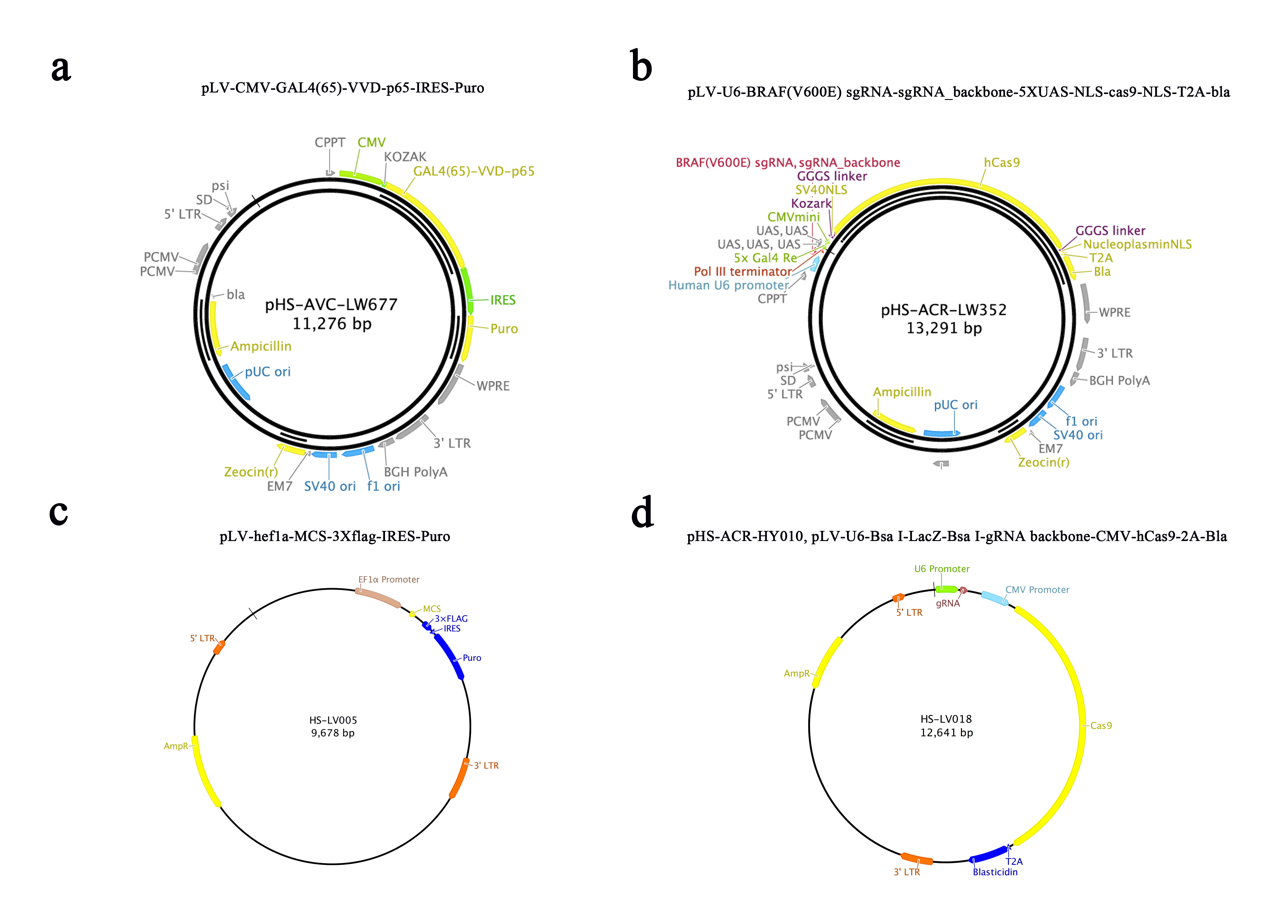

Supplement: Supplementary file 1 [file Table_1.DOCX]
